# Psychedelic integration beyond the verbal: participatory-imaginal processing as a hypothesis-generating therapeutic framework

**DOI:** 10.3389/fpsyt.2026.1895181

**Published:** 2026-08-12

**Authors:** Anthony L. Back, Robert Bosnak

**Affiliations:** 1 University of Washington School of Medicine, Seattle, WA, United States; 2 State University of New York Upstate Medical University, Syracuse, NY, United States

**Keywords:** cancer, embodied imagination, imaginal, integration, predictive processing, psychedelic therapy

## Abstract

Psilocybin therapy in patients with cancer commonly produces vivid embodied and imaginal experiences that resist verbal description, yet most current integration approaches rely primarily on cognitive and verbal processing. We hypothesize that an approach to integration that directly engages this embodied register, without requiring translation into a verbal narrative, will enable patients to re-access salient aspects of their psychedelic experience and consolidate them into a durable, re-enterable touchstone. We call this mode of processing *participatory-imaginal processing*. Drawing on Embodied Imagination, predictive processing models, and clinical observations from psilocybin therapy, we describe how participatory-imaginal processing can be invoked during integration sessions, how it provides an opportunity to revisit somatic, affective, and imaginal material from the psychedelic experience, and the way it can function as a hypothesis-generating therapeutic framework. Our method is called the DREAM protocol (Drop In, Recount, Enter, Amplify, Merge), which we designed as a structured clinical heuristic that operationalizes our approach in a form that can be taught and evaluated for feasibility and fidelity. We hypothesize that using DREAM to activate a participatory-imaginal mode of processing in integration may help patients re-engage somatically salient aspects of their psychedelic experience and identify embodied touchstones that remain accessible long afterward. We illustrate the framework using clinical material from Bosnak’s work and spontaneous reports from participants in completed psilocybin trials. Two pilot studies currently in progress are designed to assess the feasibility, tolerability, and preliminary therapeutic relevance of this approach in the immediate post-psilocybin period.

## 1 Introduction

In our clinical trials of psilocybin therapy for cancer-related anxiety and depression (1, 2), study participants commonly report vivid somatic experiences that resist straightforward verbal description. One participant recalled: “I felt what it feels like to *feel* everything; all at once. I felt my bones dissolve and my body became liquid…” He added: “I did some journaling during the trip and it was a little difficult to write because the ink was dancing on the paper as I wrote the words. Time became limitless and I felt like what it feels like to be infinite.” Another participant, who came to our retreat after a grueling intensive care unit admission, described her psilocybin experience as ‘profound’; the following day, in her individual integration session, she sat comfortably and silently before offering a single word: ‘safe.’ (We use ‘participant’ for people enrolled in our clinical trials, and ‘patient’ for the more general clinical case).

Yet the integration methods most commonly used after a psilocybin session, such as verbal therapies, journaling, cognitive and narrative meaning-making (3, 4), ask patients to process experiences like these primarily through verbal, propositional language. This creates a potential mismatch between their experience in the psychedelic session and the tools they are given to hold and process that experience. Many patients in our trials, and others reported in the literature, describe encounters with figures, landscapes, or presences that feel as vivid and real as ordinary perception yet are clearly not part of the physical world around them (5–9), as well as somatic experiences that are difficult to put into words. When psychedelic practitioners rely on integration methods that lead with cognitive-verbal processing, clinically important embodied experience may be lost or left unaddressed. To our knowledge, no current integration method was designed specifically to work with patients directly in this embodied register.

We hypothesize that an approach to integration that directly engages this embodied register, without requiring translation into a verbal narrative, will enable patients to re-access salient aspects of their psychedelic experience and consolidate them into a durable, re-enterable touchstone. We call this mode of processing *participatory-imaginal processing*. Our term ‘participatory-imaginal’ draws on the philosopher Corbin’s concept of the imaginal (10, 11), Vervaeke’s account of *transjective*, participatory knowing (12, 13), and its neurobiological rationale draws on Carhart-Harris and Friston’s REBUS model (14). We define these terms precisely in **Table 1** and develop the full theoretical grounding in Section 2; our purpose here is simply to state our hypothesis before explicating it in detail.

**Table 1:** Key terms used in this manuscript.

Term	Working definition	Source
Imaginal	A domain of experience that falls outside the dichotomy in which ‘objective’ means verifiable reality and ‘subjective’ means private, unverifiable experience: imaginal experiences — figures, landscapes, presences — are vivid, structured, and consequential, yet clearly not part of the physical world. Distinct from both ordinary perception and hallucination.	Corbin (10, 11)
Transjective (participatory) knowing	Knowing that arises from the dynamic coupling between an agent and its world rather than from observation alone; meaning emerges through participation and embodied engagement, and is not fully expressible as propositional knowledge.	Vervaeke (12, 13)
REBUS	‘Relaxed Beliefs Under Psychedelics’: a predictive-processing model in which psychedelics relax the precision of high-level prior beliefs, increasing the influence of bottom-up interoceptive, affective, and imaginal signals on experience.	Carhart-Harris & Friston (14)
Participatory-imaginal processing	The mode of processing hypothesized in this paper: engaging the imaginal and somatic material of a psychedelic experience directly, as a participant rather than an observer, without first translating it into verbal narrative.	This paper
Imaginal-somatic material	The felt content of a psychedelic experience — images, presences, and bodily sensations that arrive together — which patients can often access more vividly as embodied experience than as propositional description.	This paper
Embodiment (technical sense)	In Embodied Imagination, a two-part move from identifying a feeling (‘I notice a heaviness in my chest’) to becoming identified with it (‘I am the heaviness’) — the phenomenological shift from ‘I have a body’ to ‘I am a body.’	Bosnak (15–17)
Transit	Within the Amplify step, a shift from the patient’s self-perspective into the perspective of another being or thing encountered in the experience — a person, bird, tree, wave, or spirit — while somatic contact is maintained.	Bosnak (15–17)
Composite (‘touchstone’)	The product of the Merge step: at least three embodiments re-evoked and held simultaneously, consolidating into a durable, re-enterable state that patients can return to in daily integration practice. ‘Touchstone’ is the participant-facing term used in our trials.	This paper, adapting Bosnak (15–17)
Imaginal augmentation	A participatory process in which somatic, affective, and imaginal material functions as an internal scaffold that patients can revisit and re-engage repeatedly during integration; applied at the session level by DREAM and at the intervention level by the retreat structure.	Vervaeke (personal communication)

Bosnak’s Embodied Imagination (EI) (15–17) is, to our knowledge, the most developed clinical method for working with imaginal material, which is different than pure fantasy or projection. Originally developed for analyzing dreams, EI treats imaginal material as an encounter in a place with imaginal presences that can be seen or felt in the body, not as symbols to be decoded or hallucinations to be tolerated. This orientation is well matched to how psilocybin patients often describe their own sessions. EI has not previously been adapted for psychedelic integration in the published medical literature, so in this manuscript we describe our adaptation, which we call the DREAM protocol (Drop In, Recount, Enter, Amplify, Merge), and the pilot studies now underway to test it empirically.

Compared to existing published integration approaches, DREAM is designed to gently invite participants back into the imaginal, somatic space of their psychedelic experience as the initial integration move, and to postpone meaning-making. Other published integration approaches tend to move towards cognitive, conceptual, and verbal insights. Non-directive approaches tend to hold back from any directive therapist input (18) but patients in our experience interpret this behavior as them needing to produce insights; cognitive and acceptance-based approaches (CBT (19), ACT (20), Watts’s ACE model (21)) engage embodiment but route it through a specified cognitive skillset; guided imagery and hypnotherapy typically direct imagery toward a therapeutic target rather than following the patient’s own imaginal material; finally, Internal Family Systems (IFS) shares DREAM’s interest in multiple internal perspectives but organizes them around a privileged ‘Self’ energy (22, 23) (whereas EI-derived work gives no perspective priority; Section 2 develops this comparison further). What we believe is distinctive about DREAM is that it operationalizes direct, somatic engagement with imaginal material as a structured, teachable, fidelity-assessable clinical protocol. DREAM is more than a general orientation towards the body or an add-on to an otherwise verbal process.

Because DREAM asks patients to re-enter a psychedelic experience which can be emotionally charged, we also describe safeguards: screening for trauma history and dissociation vulnerability, trauma-informed pacing, and a clinical stance that imaginal material is treated as psychologically meaningful without being treated as literally real external fact, while the facilitator monitors carefully for overwhelm, escape, or dissociation. (More detail about safety procedures is described below in Section 5).

## 2 Theoretical orientation

### 2.1 The psilocybin experience as an imaginal realm

The term ‘imaginal’ was developed by Corbin to describe a domain of experience that falls outside the modern Cartesian dichotomy in which ‘objective’ has come to mean verifiable reality and ‘subjective’ has come to mean private experience inaccessible to verification, a dichotomy that leaves no place for experiences that are vivid, structured, and consequential yet not part of the physical world (10, 11). Drawing on Persian Sufi metaphysics, Corbin identified an imaginal realm that is different than either the physical world of ordinary perception or a hallucinated or fabricated fantasy. In Corbin’s imaginal realm, a subject may encounter presences, figures, or places that are experienced as vividly real, even while the subject retains an awareness that these experiences are not occurring in ordinary physical reality. Corbin felt that a subject could access experiences in the imaginal realm through ‘disciplined imagination’ and that valuable new knowledge could be revealed. We find this concept useful in describing psychedelic experiences that draw on bodily sensation, emotional experience, or autobiographical memory, as well as figures or presences.

More recently, Vervaeke has described related ideas using concepts drawn from 4E cognitive science and relevance realization theory (12, 13). He characterizes some forms of knowing as ‘transjective’, arising from the interactions between an agent and its world rather than through observation alone. In this formulation, meaning emerges through participation, embodied engagement, and dynamic coupling between subject and environment. Such experiences may involve forms of knowing that are not easily reducible to propositional cognition and may also alter the person who undergoes them. The person may be changed by what they come to know, and what they come to know happens through their embodied engagement, not from knowledge transfer of concepts or ideas (as in propositional knowledge). This line of thought draws on philosophical and embodied traditions associated with Heidegger (24), Merleau-Ponty (25), Gibson (26), and connected to empirical science by Varela (27). While Corbin’s imaginal framework and Vervaeke’s cognitive-scientific framework arise from different intellectual traditions, both offer language for describing modes of experience in which meaning appears to emerge through participation as opposed to detached reflection. Thus, we use the term ‘participatory-imaginal processing’ as a heuristic description rather than as a claim of theoretical equivalence between these different traditions.

### 2.2 How psychedelics enable participatory-imaginal processing

Brain imaging studies are broadly consistent with our hypothesis that serotonergic psychedelics temporarily alter the relationship between higher-order cognitive networks and lower-level sensory and bodily processing. A recent international mega-analysis (28) integrating eleven independent neuroimaging datasets across psilocybin, LSD, mescaline, and DMT identified a cross-drug pattern of increased functional connectivity between networks involved in transmodal processes (including narrative identity and abstract cognition) and networks involved in sensory and bodily input. One interpretation of this pattern is that psychedelics may transiently reduce the usual segregation between high-level conceptual processing and lower-level sensory-somatic signals, producing a relative flattening of the brain’s usual processing hierarchy. These findings do not directly establish participatory-imaginal processing, but they are consistent with a psychedelic-induced state in which habitual models of self and perception may become less dominant and unusual experiential phenomena may become more accessible.

Predictive processing models of brain function provide one theoretical account of how such changes become therapeutically relevant (29). In these predictive processing models, the brain is understood not as a passive receiver of sensory input but as an active system that generates top-down predictions about the world and continuously updates those predictions in response to bottom-up sensory and interoceptive signals (30, 31). Under ordinary conditions, a person’s top-down predictions are thought to exert substantial influence over perception, affective interpretation, and the experience of a stable, bounded self (32). Carhart-Harris and Friston’s REBUS model (Relaxed Beliefs Under Psychedelics) (14) proposes that serotonergic psychedelics temporarily reduce the priority that the brain gives to these top-down predictions, allowing sensory, interoceptive, and affective signals to exert relatively greater influence (also recognized with ketamine (33)). Thus, the REBUS model offers a possible account of psychedelic phenomenology that includes vivid sensations, unusual associative experiences, and temporary alterations in self-modeling. While we recognize that predictive processing models do not uniquely explain psychedelic phenomena, they appear potentially relevant to participatory-imaginal experiences.

Subsequent refinements of the REBUS framework emphasize the possibility that psychedelics temporarily alter predictive hierarchies related to the construction of the self. Letheby and Gerrans, for example, have argued that psychedelic experiences involve changes in predictive structures related to the ordinary sense of self as bounded (34). For patients facing cancer, whose sense of self is also be shaped by illness, mortality, and a foreshortened future, such temporary shifts could have psychological relevance. Empirical work by Zeifman et al. found that changes in negative self-beliefs after psilocybin were measurable at four-week follow-up and were associated with aspects of the acute psychedelic experience, although this work was conducted in healthy volunteers, not cancer patients (35). These findings do not establish a specific participatory-imaginal mechanism, but they suggest that temporary changes in self-related predictive structures may create conditions under which subsequent psychological revision becomes possible. The DREAM protocol is designed to engage this post-acute window in a structured therapeutic way, based on the hypothesis that somatic and imaginal material remains accessible during this period.

### 2.3 Patients experience the participatory-imaginal realm as ‘a different place’

Consistent with this theoretical account, many of our study participants speak of their psilocybin experience as ‘going to a different place’, or a series of places, so we often use similar language in our conversations with them (such as ‘the places you were in during your trip’). In an integration session, this kind of language lends itself easily to a perspective drawing on participatory-imaginal processing which allows a facilitator and patient to discuss psilocybin session experiences as occurring in a realm distinct from habitual consciousness that is characterized by altered bodily awareness, unusual experiential vividness, and reduced reliance on habitual cognitive framing. The clinical point is that psilocybin experiences contains forms of affective, somatic, or imaginal material that patients do not ordinarily access in everyday consciousness, and that might be re-visited and retrieved by integration methods designed to access the imaginal realm.

### 2.4 How embodied imagination works

Embodied Imagination was built on the intellectual foundations of Jung’s active imagination (36) and Hillman’s archetypal psychology (37, 38), but differs in that it emphasizes experiential engagement with dream material (15). Rather than treating dream images primarily as symbolic stand-ins for latent content, the dreamer is invited to re-enter the dream experientially, with coaching from the therapist, and to revisit the environments, sensations, and presences encountered there, and even to interact with them. In this method, the dream is treated less as a symbolic text to be analyzed and more as an experiential world to be entered. The dreamer’s body serves as the medium for the entry process: posture, temperature, pressure, breath, affect, and other felt bodily signals are tracked with precision and allowed to unfold. We refer to this material as ‘imaginal-somatic’ (reserving ‘participatory-imaginal’ for the broader mode of processing). In this framework, cognition follows embodied experience (instead of cognition suggesting what embodied experience would be ‘appropriate’), and meaning is understood as emerging through participation in the unfolding somatic experience.

**Figure 1:**
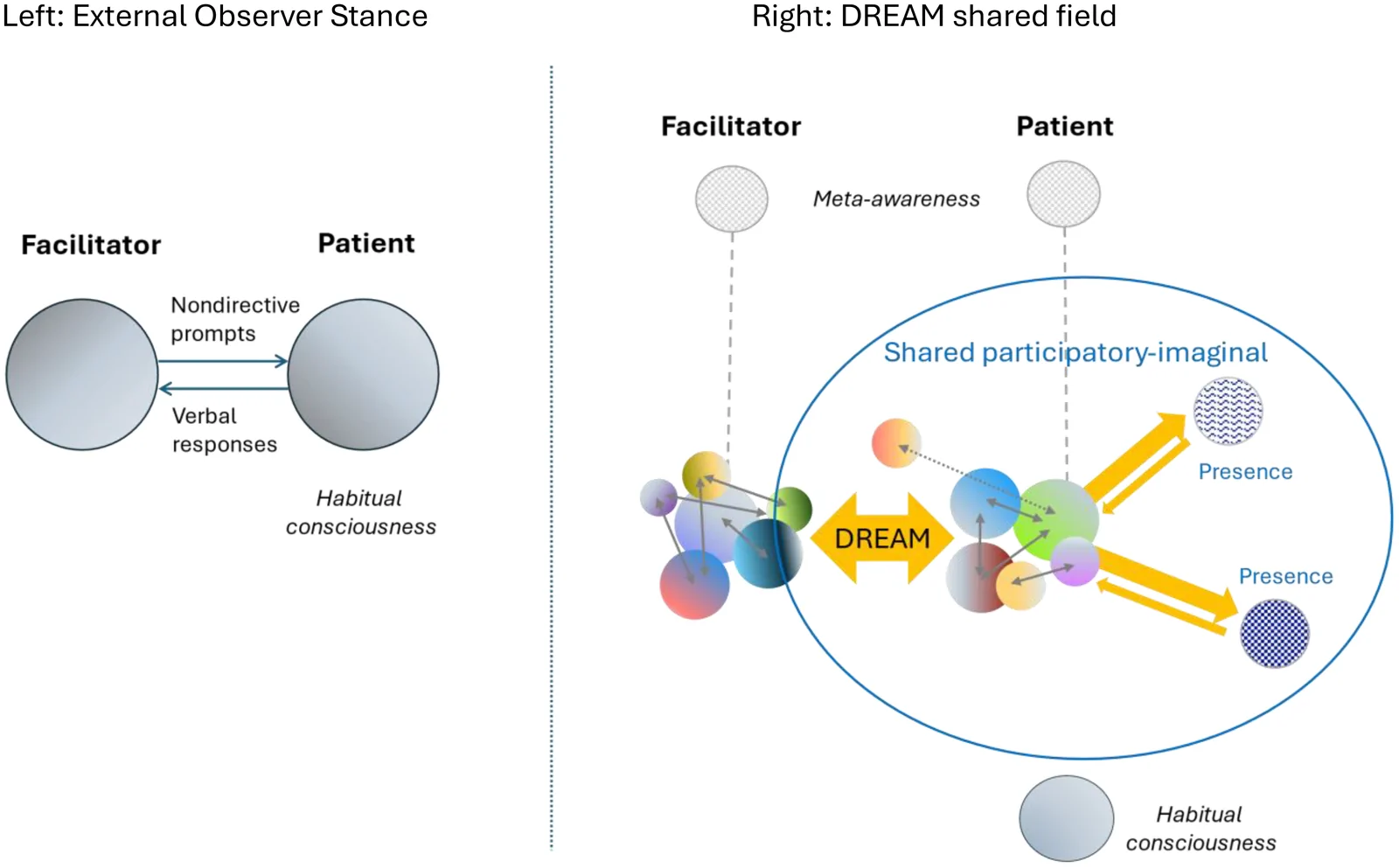
Facilitator–patient relationship during DREAM integration. **(A)** Conventional integration. Facilitator and patient are represented as unitary selves (gray circles) exchanging. The interaction occurs entirely between the habitual consciousness of the facilitator and patient. **(B)** DREAM integration. Facilitator and patient are each depicted as a self that is a complex interactive system at the edge of chaos: a decentered network of feeling-toned configurations (colored circles), each pervaded by a coherent feeling tone (denoted by color). Double-headed arrows indicate configurations in active communication within each network (the patient has one partially dissociated configuration). The DREAM process (yellow arrows) engages the two networks in their entirety, opening a shared participatory-imaginal field (large blue circle) that functions as the container for the work. The patient’s complex self has mostly entered this field because it is their integration (note that habitual consciousness is still available, lower circle); the facilitator’s self partly enters the field to accompany the patient. Within the field, the patient establishes relationships with presences — persons, animals, or features of the environment encountered in the psychedelic experience that have their own perspective and subjectivity. Single-direction yellow arrows indicate that these connections must be actively established through the DREAM process. Meta-awareness — the ongoing awareness of being in the process, analogous to the awareness of meditating during meditation — remains partially outside the shared field for both patient and facilitator (each joined to their respective system by a dashed line). The patient’s habitual consciousness (depicted as a circle largely outside the shared field) remains available but is not the mode in which the work occurs. The emergent, partially chaotic organization of the networks is meant to suggest the creativity in the integration process arising from systems operating at the edge of chaos.

Another defining feature of Embodied Imagination is that the therapist guides the dreamer to move out of their own perspective and into other perspectives within the dream. For example, a dreamer who sees a bear might first be coached to experience what it is like to see the bear from the perspective of self, initially encountering the bear as in their dream, and later, to feel the bear the way an actor becomes inhabited by the character they are playing. Thus, the dreamer could become inhabited by the subjectivity of the bear, and can feel and experience the world from the bear’s perspective. Within this experiential realm, these other perspectives, whether human, animal, environmental, or object-like, are approached as if they possess their own integrity and distinct qualities rather than as projections of the dreamer’s psyche. (**Figure 1**) The EI therapist will guide a dreamer to allow themselves to become ‘inhabited’ by different beings or elements within the dreamscape, so that embodied sensations of these beings or elements arise in the dreamer’s body as additional imaginal-somatic material. These beings or elements are treated as genuinely other since they phenomenologically present themselves as other than self, which supports a stance of curiosity, reciprocity, and ethical regard. The cumulative effect is a form of distributed subjectivity in which the dreamer’s identity becomes plural and relational. Overall, Embodied Imagination offers a method that privileges experiential immersion, somatic tracking, and multi-perspectival engagement.

In an Embodied Imagination session, these multiple perspectives are treated as a dynamic complex system composed of multiple interacting beings, affects, and embodied selves that collectively constitute the dreamer’s expanded self-experience. The embodied imagination process does not aim to subsume these figures back into a dominant ego, or translate them into a single coherent narrative, or subsume them into a larger entity, and there is no priority given to any particular perspective. EI posits that this multiplicity of contrasting embodied perspectives may create conditions with novel experiential configurations from which emerge meaning and insight. But EI’s use of multiple perspectives differs from the other well-known multiple selves approach, Internal Family Systems (IFS) (23). Unlike IFS which prioritizes ‘Self’ energy as a preferred perspective, Embodied Imagination does not prioritize any perspective. The EI “composite” of multiple inhabited perspectives is understood as shifting the organization of the complex system of the self, a reconfiguration of the complex self-system that allows meaning to emerge.

A final feature of Embodied Imagination deserves emphasis because it differentiates the method from approaches in which the facilitator is positioned as an external observer of the patient’s process. In EI, the facilitator is an engaged participant in a shared therapeutic process of attunement and experiential tracking. As the patient describes imaginal and bodily material, the facilitator can notice parallel somatic or imaginal resonances in their own bodies. Such facilitator resonances are considered to be potentially informative aspects of therapeutic attunement, while still requiring reflective judgment and clinical restraint. (Note: we have deliberately avoided the psychoanalytic term *projective identification*, which frames the phenomenon as an artifact of distorted perception, and instead use *participation* in the phenomenological sense developed earlier in this paper: a co-constituting engagement in which subject and object are mutually shaped).

### 2.5 Relevance to cancer patients

For cancer patients, the DREAM protocol may offer a new way to hold the threat to their lifespan. The day-to-day reality of oncology care emphasizes treatment protocols and adherence to treatment schedules, detection and management of physical symptoms, and often an imperative of ‘doing everything possible,’ ‘doing things correctly,’ and searching for new information. DREAM works in a different register than biomedical information: rather than reemphasizing illness- and treatment-related concerns, re-entering the imaginal-somatic material of a psilocybin experience could allow other aspects of the patient’s life, such as relationships, life goals, and spirituality, to become relevant again. A recent interpretive phenomenological study of psilocybin-assisted psychotherapy in patients with life-threatening illness, predominantly cancer, illustrates what such a shift can look like: before treatment, participants equated death with physical obliteration, described an ‘obsessive focus’ on death, experienced the future as an ‘agonizing wait,’ and the present as ‘endless marking time’; among those who described their treatment as successful, these themes did not give way to avoidance of death but instead, death became ‘also part of life,’ the future ‘opens up with hope,’ and the present rejoins ‘the flow of life.’ (39) The investigators characterized this transformation as an inversion in where patients stand in relation to their own lives — from observer to participant — and found the psilocybin necessary but not sufficient to produce it, findings that converge with the participatory frame developed here and with the premise that the integration component deserves deliberate design.

## 3 Adapting embodied imagination for psilocybin integration

Embodied Imagination was developed and refined by Bosnak over five decades of clinical experience, which included his own 1970s training in LSD psychoanalysis at a psychiatric hospital in Zurich with a Sandoz LSD license. He developed EI to work with imaginal material surfacing in dream states. Since then, dream states and psychedelic states have been proposed to produce related phenomenology, including vivid internally generated imagery, altered self-processing, and reduced top-down cognitive control, through different neurobiological mechanisms (40, 41). This preliminary evidence and our experience that dreaming and psilocybin share this relaxation of top-down constraint with increased bottom-up signaling is what led us to adapt EI techniques to post-psilocybin integration. We do not claim psilocybin states are equivalent to dream states; the experiential, neurochemical, and contextual differences are substantial. But since EI’s core mechanism, engaging imaginal-somatic material through embodied tracking rather than verbal interpretation, takes advantage of the brain’s relaxed top-down state produced in either dreaming or psilocybin sessions, EI seems well suited for psychedelic integration.

However, while existing accounts of EI are clinically generative and phenomenologically detailed, EI has not been well studied empirically. Szuster conducted a pilot study examining the use of EI in the creative process (42), and Lawson conducted a small study of EI for patients with serious illness as an unpublished PhD thesis (Lawson, personal communication), but no other studies examining psychometric outcomes with dreamers have been conducted. So we have adapted EI into a form that addresses psychedelic integration specifically, recast its essential elements into an approach that could be taught with consistency, and delivered with reasonable fidelity so that it can be evaluated with measurable outcomes, without oversimplifying it or losing flexibility to respond to the wide scope of experiences that occur in psilocybin sessions.

**Figure 2:**
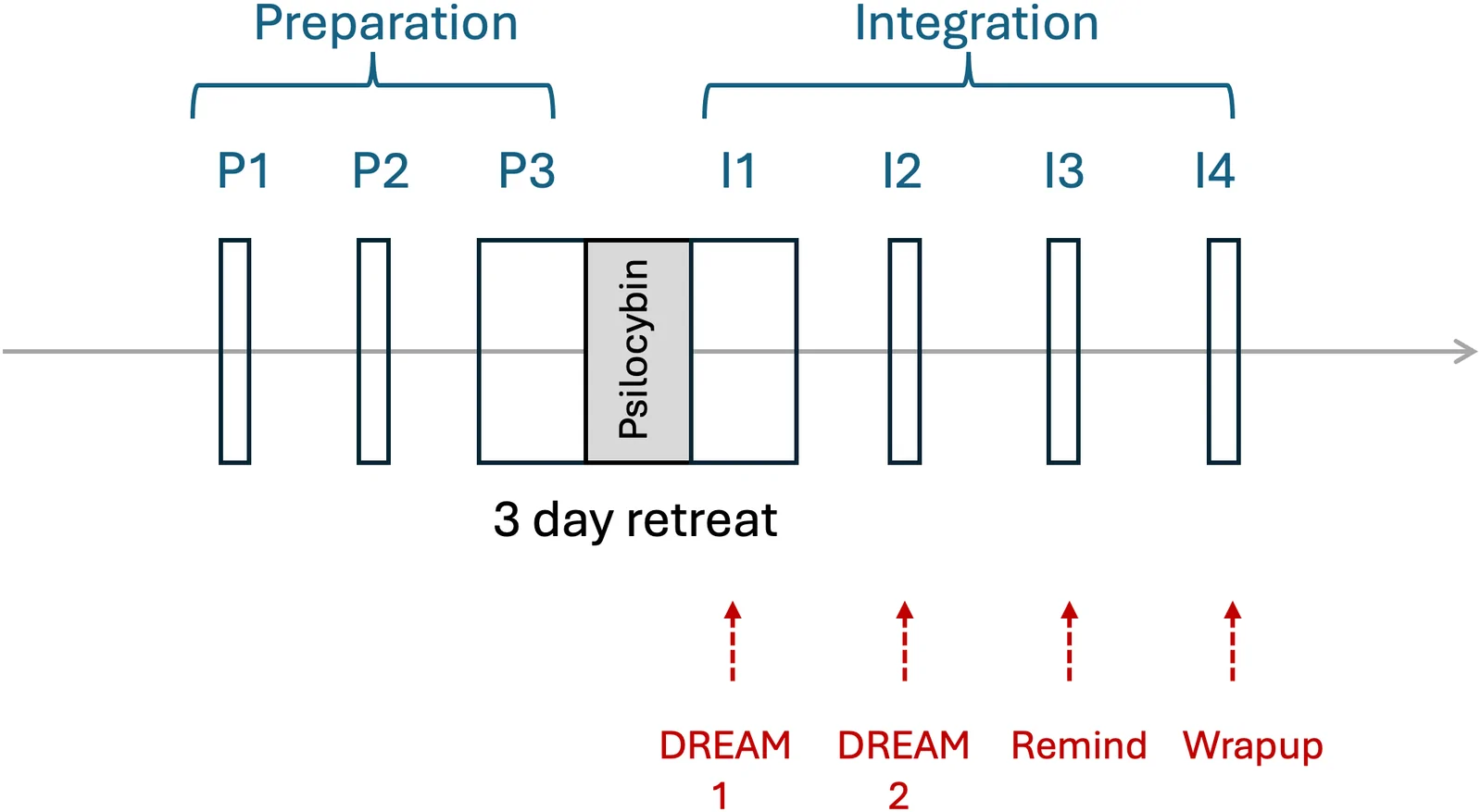
Timeline of group retreat psilocybin therapy and DREAM activities. P1, P2, P3 are Preparation sessions, all 1 week apart. I1, I2, I3, I4 are Integration session, all 1 week apart. DREAM sessions 1 and 2 are included in the Integration sessions.

In adapting Embodied Imagination, we have preserved the clinical stance of the therapist/facilitator while creating a procedural scaffold as a heuristic to follow. We defined key elements of EI’s clinical stance, including the imaginal realm as a distinct mode of experience and knowing, the emphasis on embodied sensations, and the use of a composite of sensations as a reflection of a complex self, which are retained intact. What we modified were procedural elements that adapt EI to the psilocybin-integration context. To do this, we conducted a task analysis of EI as practiced by Bosnak, identified the facilitator (or therapist) moves that define the method, and the milestones that govern how the moves are sequenced. This led to three significant adaptations. First, we shifted the induction frame from hypnagogia, which implies a sleep-like state suitable for dreaming, to ‘re-entering your psilocybin trip.’ Second, we shifted the pacing to allow for faster entry into the psilocybin experience, because we find that the re-entry state is easily accessed soon after a psilocybin session. Third, since we plan to use this in a group retreat intervention, we have designed into the retreat individual sessions for patients to re-enter their trip with just a facilitator pair, and later a group session for patients where they can contribute whatever portion of their embodiments they are comfortable sharing. (**Figure 2**)

The resulting five step heuristic, DREAM (Drop In, Recount, Enter, Amplify, Merge) serves three purposes: to scaffold facilitator training, to support facilitator use of the technique, and to enable fidelity assessment through observable markers tied to each step. From this heuristic we wrote a detailed treatment manual. The manual was refined through an internal, iterative manual-development process: informal practice sessions (AB) were audio-recorded solely for supervision by Bosnak (RB) and manual development. These sessions were not designed or analyzed as a research study, and no participant-level data, quotations, or findings from them are reported in this manuscript. The University of Washington Human Subjects Division reviewed this activity and determined that it did not constitute human subjects research. Using Bosnak’s supervision feedback, we have revised the suggested opening language for each step, specified where flexibility to the patient is important, and added additional material (especially in the Amplify step). We are now conducting a separate IRB-approved pilot feasibility study with 10 patients who have had a psychedelic experience not longer than 3 months prior to enrollment, that includes patient feedback on the heuristic and their experience in the sessions, and qualitative comments on what emerged that they found to be valuable. Our intent for this initial protocol is to evaluate its feasibility and safety for post-psilocybin integration, and to use the results to adapt the DREAM protocol so that facilitators can be trained to deliver it with reasonable fidelity. We expect further evolution to occur as facilitator experience and empirical data accumulate.

## 4 The five steps of the DREAM protocol

The five steps of DREAM are: 1) Drop in, 2) Recount, 3) Enter, 4) Amplify, and 5) Merge. After the psilocybin session, each patient will have two individual sessions (the first session at the retreat, the second session 1–2 weeks later on zoom), with each session lasting 45–60 minutes. (**Table 2**).

**Table 2:** DREAM protocol framework.

DREAM step	Facilitator behavior	Hypothesized process	Observable fidelity marker	Safety/titration rule
Drop In (8–10 min)	Invites the patient to close their eyes and settle into embodied, mindful presence using somatic grounding, breath awareness, and body awareness; slow pacing, quiet voice, minimal visual stimuli (“Just allow yourself to be completely in your body.”)	Shifts attention away from the exteroceptive predictions that dominate ordinary waking consciousness and toward interoceptive signals, loosening reliance on habitual self-narratives.	Facilitator maintains slow pacing and quiet voice throughout the step; patient’s language shifts from exteroceptive/environmental references toward bodily/interoceptive references.	If the patient cannot settle or shows signs of distress, extend grounding before moving to Recount; do not rush the transition.
Recount (8–10 min)	Invites the patient to re-enter and re-experience their psilocybin session in the present tense; asks body-location questions (e.g., “Where in your body are you feeling that now?”).	Reactivates embodied and affective memory of the psilocybin experience via state-dependent retrieval; engages insula-mediated integration of interoceptive experience and bodily memory.	Patient speaks about the experience in present tense; facilitator asks at least one body-location question per notable feeling reported.	If recounting produces overwhelming affect without a somatic anchor, pause and return to Drop In grounding before continuing.
Enter (10–12 min)	Follows the patient into a specific somatic feeling using Bosnak’s two-part technique (identify the feeling, then become identified with it); chooses a first embodiment that is low-threat.	Sustained attention increases the salience and granularity of the interoceptive signal and delays habitual interpretive/narrative processing — the shift from ‘I have a body’ to ‘I am a body.’	Facilitator holds a single embodiment for an extended period without shifting to interpretation; patient’s language shifts from having a sensation to being/identifying with it.	First embodiment is deliberately chosen to be low-threat; if intensity rises too quickly, slow further or shift to a neutral bodily anchor.
Amplify (12–15 min)	Follows the patient through further re-experience; identifies at least 2 additional embodiments; invites ‘transits’ into the perspective of another being or thing encountered.	Transits surface previously unavailable material (images, memories, affects) consistent with reduced top-down constraint and increased sensitivity to bodily and affective signals.	At least 2 additional embodiments identified; at least one transit attempted; pacing remains slow and unhurried throughout.	Embodiments are sequenced from low-threat to more charged; if a transit becomes overwhelming or triggers dissociation, ground back to the body before continuing.
Merge (5 min)	Directs the patient to re-evoke at least 3 embodiments sequentially, then hold them simultaneously as a composite; provides home-practice instructions.	Holding multiple embodied states simultaneously consolidates them into a durable, re-enterable composite (‘touchstone’) available for home integration practice.	Patient identifies and holds at least 3 embodiments simultaneously; facilitator gives explicit home-practice instructions (~2×/day, including before sleep).	If composite holding produces distress rather than integration, reduce to fewer embodiments or a single grounding embodiment before ending the session.

1) Drop in (8–10 min). Establish the patient’s somatic presence. We ask the patient to close their eyes and feel their embodied, mindful presence, using somatic grounding, breath awareness, and body awareness. (“Just allow yourself to be completely in your body”).

The Drop in step begins with exteroceptive awareness (surface sensations at the skin, the external world, the room), then moves to interoceptive process (sensations below the surface of the body). While we focus on the here-and-now, this step differs from a typical mindfulness body scan because it moves towards interoceptive awareness using the patient’s hand as a cue. From a predictive-processing perspective, the Drop In step can be understood as encouraging a shift of attention away from the exteroceptive predictions that often dominate waking consciousness and toward interoceptive signals arising from the body. This shift may allow processing in which bodily sensations become more available for exploration, loosening reliance on habitual narratives about the self (43, 44). The slow pacing, quiet voice of the facilitator, the reduction in visual stimuli are important to create space for the interoceptive signals, which are less granular and less familiar, to be felt.

2) Recount (8–10 min). Invite the patient to approach and re-enter their psilocybin trip. “Now see if you can re-enter your trip, and allow yourself to re-experience it. Tell me what you are feeling and experiencing. Speak in the present.”)

The Recount step is intended to reactivate aspects of the embodied and affective memory of the psilocybin experience. When patients re-enter their psilocybin trip through their interoceptive experience, they are doing something different than cognitive recall. Memory is state-dependent, meaning that information encoded to memory in a specific physiologic state is easiest to retrieve when the brain is in that same state or a similar state (45). The insula has been proposed to play an important role in the integration of interoceptive experience and bodily memory (46, 47). When the facilitator asks the patient to pause with, ‘Where in your body are you feeling that now?’, the question can help the patient re-engage bodily aspects of their psilocybin session experience. This may facilitate partial reactivation of neural and physiological patterns associated with the psilocybin session.

3) Enter (10–12 min). Accompany the patient as they re-enter and re-experience their trip. (“Could we pause on that feeling of heaviness in your chest? Let’s just hang out there for a few breaths. That heaviness may change, or may not.”) This is the entry into imaginal-somatic material by way of somatic memory, through a process of interoception in which the patient re-visits their psychedelic experience and locates, with the facilitator’s guidance, somatically vivid embodied sensations that can serve as touchstones later. Note that the facilitator chooses a moment to build a first embodiment that is low threat, to reduce any perceived barriers to entering the imaginal realm.

By inviting the patient to go more deeply in a specific somatic feeling (to locate it, notice its texture or quality, track movements), the facilitator is inviting sustained attention to bodily sensations, which can increase the relative salience of interoceptive signals. The patient is guided to attend closely to feelings that are present in the moment rather than jumping into habitual interpretations and old narratives. By holding the patient to notice the raw interoceptive, bottom-up signals and holding them back from moving directly into their usual labeling, interpretive, and meaning-making cognitive processes, this slower pace may allow bodily sensations to be explored in a way that delays or derails habitual explanatory narratives. Bosnak uses a two-part technique: to start by identifying the feeling (“I notice a heaviness in my chest”), then becoming identified with the feeling (“I am the heaviness, I am dense, warm, pressing”). This is the phenomenological movement from ‘I have a body’ to ‘I am a body.’ The practical effect of this two-part approach is to increase the patient’s attention on the bottom-up feeling and then to increase the granularity of the feeling so that the patient is feeling the intrinsic qualities of the heaviness, not elaborating a narrative that treats the heaviness as an external object. In this example, the ‘heaviness’ is considered to be a single embodiment encompassing observer and observed.

4) Amplify (12–15 min). Follow the patient as they re-experience their trip, and pause to identify at least 2 more embodiments. The facilitator can also invite the patient to ‘transit’ from their self-perspective to the perspective of another being they encounter, which could be a person, a bird, a tree, a boat, a wave, or a spirit. (“Can you approach the tree, moving right up next to it, feel the bark, smell the leaves. [pause] Now as you feel the bark, can you sense the life of the bark? Is there a life energy moving in the bark?”). In a transit, the patient enters a state where they feel both what the other being is feeling, which Bosnak describes as the other as ‘coming to inhabit’ the patient, but without the patient losing their own sense of self. Typically, Bosnak recommends that after the first embodiment, the next embodiments are more unfamiliar, possibly charged, and even threatening (for example a presence associated with fear).

When the patient is in a transit into the perspective of the other, something unexpected and new will often arise – an image, a memory fragment, a new bodily feeling, a new affect – and these may seem illogical or not obviously related. However, we interpret the appearance of new images, affects, memories, or bodily sensations as material that was not previously in awareness. The phenomenology of transit experiences is broadly consistent with what predictive-processing theory would predict under conditions of reduced top-down constraint and increased sensitivity to bodily and affective signals. The process must proceed slowly, because moving quickly will trigger reinstatement of habitual top-down predictions.

5) Merge (5 min). The facilitator directs the patient to re-evoke, sequentially, at least 3 embodiments from the session. Each embodiment is held for a moment, just long enough for the patient to recognize it again. Then the facilitator asks the patient to ‘try to hold all three embodiments simultaneously’, in a ‘composite’ embodiment. (“Let’s see if we can feel the heaviness in the chest, the bark of the tree, and the freshness of the sky—all at the same time.”) This last step, which re-evokes the 3 embodiments briefly, is the ‘touchstone’, or ‘composite’. The recommendation is for the patient to briefly revisit the 3 embodiments and the touchstone as the patient’s integration practice, for a few minutes twice a day (with the second time in bed just before falling asleep).

When the patient holds multiple embodied states simultaneously (the heaviness in the chest, a warmth in the belly, the feeling of being in the ocean on the legs; three is a practical number to aim for), they are held simultaneously in awareness without being immediately organized into a single narrative frame. From a predictive-processing perspective, this process might be understood as temporarily maintaining multiple competing interpretations or experiential signals without rapidly resolving them into a single dominant model. The composite, or ‘touchstone’ that emerges sets the stage for new feelings, ideas, or insights to arise that would not have followed if only one of the embodied states was recalled. A neuroscientific analogy for this strategy is found in the creativity literature, where research documents that a new insight emerges when the brain maintains competing activation patterns while avoiding premature closure (48). The act of holding multiple embodied states simultaneously may create conditions in which novel insights become more likely to emerge than if only a single embodied state is recalled. The composite is an interoceptive synthesis that is located in the body instead of in propositional memory.

The temporal structure of a DREAM session differs from that of the original psilocybin session. A DREAM session does not replay the entire psilocybin session in clock time: a 45–60 minute DREAM integration session revisits a psilocybin experience that unfolded over roughly six hours, and it does so non-chronologically, following somatic salience rather than narrative sequence.

The DREAM protocol requires that the facilitator maintain a state of not-knowing and suspend temporarily their habitual professional knowing. As the facilitator guides the patient into the imaginal realm, they often generate ideas about how the patient might cope better, or needs more self-compassion, or should rewrite the narrative of themselves as a cancer patient—but instead of acting on those hunches, the facilitator maintains (and thus assists the patient to maintain) the state of non-judgmental not-knowing so they can follow the patient’s lead. The DREAM facilitator’s stance of postponed knowing shares with the non-directive approach a refusal to impose interpretive content on the patient’s experience, but DREAM facilitators work within a shared imaginal field, tracking somatic shifts, and inviting transits and embodiments, setting up conditions that allow for emergence of meaning. Bosnak likens the facilitator to a carpenter who knows how to use hammers and nails (the tools needed to build scaffolding) but does not know what the house will look like.

Where does this re-entered state sit on the continuum spanning ordinary waking consciousness, dreaming, and the psilocybin state? Whereas during a psilocybin session a patient’s top-down prior predictions (everyday habits of mind) are significantly relaxed and bottom-up (imaginal and interoceptive) signals are given more priority, a DREAM session has similar characteristics but at considerably lower amplitude. Relative to dreaming, in a DREAM session the patient retains volition, meta-awareness, and dialogic contact with the facilitator. This positioning has a practical consequence for manualization: the facilitator’s task is to help the patient find a productive zone, deep enough that imaginal-somatic material is vivid, awake enough that dual awareness is preserved. The pacing, grounding, and titration procedures described above are the operational means of doing so.

## 5 Safety considerations

Re-entering a psilocybin experience through DREAM could potentially surface traumatic memory, somatic distress, or affective flooding, so the pilot trial protocol builds in explicit safeguards for eligibility, monitoring, and pausing or stopping.

*Contraindications*. The pilot study excludes individuals with a history of persisting perceptual or dissociative symptoms following prior psychedelic use, including Hallucinogen Persisting Perception Disorder (HPPD) or persistent depersonalization/derealization. Individuals with current evidence of psychosis, significant dissociation, or persisting distress are also excluded at screening. The feasibility pilot (BACK009) is meant to establish feasibility and safety in a carefully screened, low-risk population before the protocol is extended to populations that might include psychosis vulnerability.

*Screening.* Before beginning DREAM, facilitators screen for the contraindications above and make an informal assessment of what we call somatic literacy: whether the patient can tolerate an invitation to attend to bodily experience without distress. This is typically assessed through a direct question, such as, “If I invite you to go back into your session and feel into your body, is that okay — does that create any problems for you?”

*Informed consent.* Because DREAM invites re-entry into material that can be emotionally charged, informed consent should go beyond generic psychotherapy consent to disclose the specific risks of psychological distress and of re-encountering traumatic material. The consent documents for our feasibility pilot (BACK009) disclose these risks explicitly.

*Facilitator training and in-session monitoring*. Facilitators are trained to recognize signs of withdrawal, overwhelm, or dissociation, including flight, fight, or freeze responses, and to titrate the depth of somatic engagement accordingly: slowing the pace, returning the patient to grounding, and prioritizing stabilization over continued progression through the DREAM steps. The Merge step is held lightly: if a patient cannot sustain multiple embodied states without distress, the facilitator works with whatever the patient can hold comfortably.

*Stopping rules*. Within a session, facilitators pause and return to grounding whenever a patient shows signs of withdrawal or overwhelm. If a patient develops strong affect that is difficult for them to regulate, or shows clear signs of dysregulation (flight, fight, or freeze), a harder stop, including ending the session, would be indicated.

*Modification for trauma history and dissociation vulnerability.* For patients with a known trauma history or dissociation vulnerability, facilitators monitor the Amplify step’s transits with particular care, since transiting into the perspective of another being or thing can be more likely to surface intense affect. At any suggestion of distress, panic, or freeze during a transit, the facilitator stops and returns the patient to grounding.

*Modification for high illness burden*. Sessions are adapted to the patient’s available energy; where illness burden or fatigue warrants it, session length may be shortened, prioritizing the patient’s capacity over completion of all five steps.

*Referral pathways*. When material surfaces that warrants care beyond the scope of the facilitator relationship, participants will be referred to a mental health professional with relevant expertise (for example, a clinician with expertise in persisting effects of psychedelic use or severe trauma if this were to surface during the session).

*Suicidality monitoring*. This safeguard is required for IRB-approved clinical designs so is incorporated into the pilot feasibility study before the first session begins.

## 6 Illustrative clinical anecdotes

While we do not yet have reports for clinical trial participants specifically testing the DREAM protocol, we do have clinical anecdotes that illustrate this process, both from Bosnak’s extensive clinical practice of dreamwork and Back’s completed trials. Bosnak documented a case study, using dreamwork only, involving a Japanese cancer patient with a large anal tumor who was withdrawn and unreachable (15). The physician on the unit said “When he was admitted to our unit [ … ] we felt a solid barrier around him [….] He withdrew into his hard shell.” The only dream that the patient could remember since being diagnosed with cancer featured a cat: “I am in a strange room in a city. I’m lying on a couch. In the back of the room [ … ] I see two lights. At first I think it is the traffic outside. But then I realize it is a cat. I see the cat move around the room.” Bosnak describes using embodied imagination to ‘help him feel back into the dream [….] The dreamer observes with pleasure how graciously the cat moves. He loves cats [….] I encourage him to feel into his body and feel it mimic the movements of the cat.” Later: “He observes the sleek, supple movements of the cat, and for a brief moment he can feel a loosening in his body.” At a subsequent session, the dreamer says “I am not the cat.” Bosnak replies: “of course you are not the cat [….] But it is possible to feel into the life force of the cat by observing the cat.” And then: “when we look at the cat this time, he can feel the movement of the cat throughout his body. A blissful expression comes on his face.” The dreamer shifted from the habitual consciousness of his immobilized body into the supple body of the cat. The dreamer, who in the West might have been diagnosed with demoralization syndrome, appeared to regain a sense of agency, and was subsequently able to make medical decisions and connect with his family. We interpret this as an instance of a spontaneous transit, identification with another being’s felt experience, occurring alongside the dreamer’s apparent shift in agency; whether transits reliably predict such shifts is an empirical question for future study, not one this case can answer.

Post-psilocybin experiences from Back’s completed clinical trials (1, 2) suggest that occasional patients enter a participatory-imaginal mode of processing spontaneously. We present 3 themes, all illustrated with quotes from the same participant, and then return to the two participants mentioned in the introduction. We note that many others in the clinical trials did *not* have experiences in a participatory-imaginal mode, so we regard these anecdotes as case studies.

*Spontaneous entry into participatory-imaginal processing.* “It’s like the first time in my life I’m not able to say what I’m thinking….As I was sinking in, I had this thought of, don’t you know who I am? I’ve been here forever.” Next, this participant found themselves in a series of places: “I saw the house where I was assaulted as a child. I went in and I saw what happened….Then suddenly I’m in a hospital room seeing myself die…” We interpret this as spontaneous entry into a participatory-imaginal mode, unprompted by any structured facilitation indicating that this mode of processing can be available. Whether the prompting we designed in the DREAM protocol is a reliable route is again a question for future study.

*Identification of imaginal-somatic material*. “I called my husband to me and I let him sit in my heart. I just felt this love. The love he has always had for me.” Months after her psilocybin experience (our formal followup ended at 6 months), this participant could still return to this embodied feeling of love. We interpret this persistence of an embodied feeling, recoverable months later, as an example of what we term a touchstone; whether touchstones built through invited, structured re-entry are more durable than this spontaneous one is a question for further study.

*Emergence of new ways of living with mortality*. “It was like a slap upside the head. I was like, dummy, you need to receive. So I was like ok, I will receive this….I felt like when I could receive, I will release … I can trust and let my body do its thing. My body is still beautiful and working, even though there are things [cancer] happening inside of it.” Later she reported “I freaked my husband out because I told him I saw my own death. And I was like no, not now. No, really. Don’t let go so fast.” We interpret this as new meaning emerging from embodied sensation, illustrating the shift from propositional to participatory-imaginal processing; whether this kind of shift is reliably associated with measurable change is a question for future studies, not one a single case can establish.

The participant quoted in the introduction, who could *feel* everything all at once, at the opening of his trip, later felt his body become “liquid and the whole world was liquid and full of color.” His sensations then included the following: “I felt and visualized my jaw dissolve on several occasions and realized I was clenching my jaw and I let go and for the first time I felt like what it means to “let go.” Later, he went to the bathroom “and when I was peeing the toilet was full of jellyfish and I realized I could pee jellyfish. They were so beautiful and translucent and colorful and moving in harmony. I had no idea there was so much power in the mind.” Near the end of his telling: “For a period of time nothing made sense and it was so funny to realize how absurd it was to make sense of things. I felt like a billion colors. And I felt love. So much love like I’ve never felt before. I objectified my cancer and wanted to be angry at it and yell and scream and then started laughing at how absurd that was. So I gave it a hug and loved and forgave. I wrestled with the idea of death and it didn’t seem possible to die. It felt like my body could die, but my feelings could never die and, I know this sounds silly but I realized that death is love.” We interpret this extended sequence as spontaneous participatory-imaginal processing sustained across the session and reported later unprompted by a facilitator debriefing, suggesting that this mode of processing is available.

The other participant quoted in the introduction who did not have words initially, later said “I just saw lots of lines, and I felt warm and comfortable and cared for and loved” which she compared to “it was like getting warm and sunny, I felt like a cat lying in the sun.” After a stay in the intensive care unit that she described as ‘traumatic’, she said “I feel calm and safe and grounded. It’s sort of like all-encompassing.” And later in this integration she said “I think this calmness is healing the trauma. I’m not quite able to describe it or pin it down, but I think this is what I need.” An embodied feeling that persisted was “hearing [my spouse] crying” and feeling her spouse “letting herself be held” from which emerged a “safety that extends to everyone. It’s not just me that’s safe. It’s my kids and [my spouse].” She said “what I want to use to help me is to remember the feeling that the heart opens and closes. So when I get frustrated or upset, I remember that the heart opens and closes. My intention is to keep my heart open in all of my interactions with the world, but the heart opens and closes.” We interpret the word ‘safe,’ and the recurring image of the heart opening and closing, as touchstones this participant generated spontaneously. Whether the structured DREAM protocol, with its emphasis on finding touchstones, produces more durable or more accessible touchstones is a question for further study.

## 7 Pilot empirical studies using the DREAM protocol

The participatory-imaginal approach described here occupies a distinctive position in the current landscape of psychedelic intervention design. Industry-led trials have largely adopted a pharmacology-first paradigm with minimal psychotherapeutic content (49); other major programs use non-directive psychological support designed to minimize therapist influence on the experience (18) and a smaller body of work has paired psilocybin with manualized cognitive or acceptance-based therapies including CBT (50) and ACT (20). Watts’s ACE model (Accept, Connect, Embody) foregrounds embodiment as a core component of psilocybin integration, although ACE does not specify how facilitators engage embodied material in session (21). Each of these designs reflects a substantive theoretical position about what produces therapeutic benefit: pharmacological action alone, an unstructured experience with a non-directive debrief, or a post-psychedelic integration using a specific cognitive skillset. The framework presented here proposes that participatory-imaginal processing may represent a primary therapeutic substrate worthy of deliberate engagement, and that imaginal augmentation (a term suggested by Vervaeke, personal communication) can function as a participatory process in which somatic, affective, and imaginal material can be revisited and re-engaged repeatedly during integration, and DREAM provides a specifiable, teachable, and empirically testable mechanism for doing so.

Two trials currently in progress are designed to test this proposition. BACK009 is an IRB-approved feasibility pilot of the DREAM protocol as a stand-alone integration intervention in adults with a history of psilocybin or other psychedelic experience. The protocol pre-specifies three primary feasibility outcomes, each with a success criterion set in advance of data collection: session completion rate (percentage of enrolled participants completing all three intervention sessions; success criterion ≥ 80%), intervention satisfaction (a 10-item questionnaire assessing satisfaction, perceived helpfulness, and likelihood of recommending the intervention, rated on a 5-point Likert scale; success criterion: mean ≥ 4.0), and protocol fidelity (percentage of protocol steps completed per session, documented on fidelity checklists; success criterion ≥ 85% adherence). Secondary outcomes include participant tolerability, the emergence of touchstones in post-session documentation, Psychedelic Insight Scale (51), and Psychedelic Experienced Integration Scale (52). The trial uses semi-structured interviews after the integration sessions to characterize the participatory-imaginal processing mode in patients’ own descriptive language, to provide qualitative feedback needed to refine the protocol before deployment in larger trials.

In our Group Retreat Psilocybin Therapy trials, the retreat itself is a deliberate application of imaginal augmentation at the level of the whole intervention (43). The retreat enacts the three-part structure van Gennep identified in rites of passage (53) (separation, liminality, reincorporation) with Turner’s elaboration of the liminal phase (54) framing the psilocybin session and its integration (55). From the participatory-imaginal perspective, this ritual container is meant to intensify and organize embodied, symbolic, and relational processes in ways that support psychological change. The layering is intended to be synergistic: the retreat provides a macro-frame for participatory-imaginal processing over weeks, while a DREAM session provides a micro frame over the course of an hour, translating psilocybin experiences into touchstones that patients can return to in daily life. This remains a theoretical proposition rather than an established mechanism, and the current designs cannot separate DREAM-specific effects from effects of the retreat container (see Limitations).

BACK007, a Phase 2 group retreat study in patients with metastatic cancer and symptoms of anxiety and depression, will test the full integrated framework: a preparation incorporating interoceptive attunement training, a three-day retreat structured as macro-augmentation, and DREAM as the integration methodology operating across the post-session window. Outcomes include the Hospital Anxiety and Depression Scale (56), five MAIA-2 subscales (57, 58), the Mystical Experience Questionnaire (59), and a measurement battery designed to detect the perspectival and participatory dimensions of change. Together these trials are intended to provide the empirical foundation for the framework presented here. Prior trials have established early evidence that psilocybin produces benefit for cancer patients with distress (60, 61). These pilot studies ask whether deliberately scaffolding participatory-imaginal processing through coordinated somatic preparation, use of a ritual container that affords imaginal augmentation, and DREAM integration can produce measurable outcomes that seem worth testing against existing worth testing against minimal guide support and non-directive integration approaches.

## 8 Discussion

The contribution that the DREAM protocol potentially makes to psychedelic integration is a structured way of working with embodied experience toward therapeutic ends. DREAM can be distinguished from existing approaches in three respects. DREAM is less dependent on verbal processing than cognitive or acceptance-based integration models: it stays within the somatic and imaginal register the psychedelic experience itself often arrives in, rather than translating that material into propositional language before working with it. The DREAM process starts with embodied experience, and the DREAM approach holds that experience as open and porous, allowing meaning to arrive later, if it arrives at all, from the patient’s own process instead of the facilitator’s framing. Finally, DREAM does not require patients to arrive at explicit “meaning” from their experience at all, a demand that other integration approaches often carry implicitly, and one that can itself become a source of discouragement, or feel like a failure, when insight does not come readily, particularly for patients confronting a life-threatening illness.

The framework described here generates three core predictions. First, that the participatory-imaginal realm can be reliably accessed through structured facilitation, that using the DREAM protocol can enable most patients to re-access imaginal-somatic material from their psychedelic experience. Second, that this access adds to a patient’s integration and to the eventual positive impact of the psychedelic experience. Third, that accessing the imaginal realm through DREAM does not add to the risks of a psychedelic experience or introduce new kinds of harm. The feasibility pilot described in Section 7 (BACK009) is designed to provide preliminary, hypothesis-generating evidence on all three: completion and fidelity data bear on reliable access, the secondary outcome measures and qualitative interviews bear on integration benefit, and adverse-event monitoring bears on added risk. But a single-arm pilot of this size cannot establish any of these predictions definitively, and at best can indicate whether DREAM is worth testing further.

The three core predictions each suggest specific forms of counterevidence to the use of DREAM. Evidence against reliable access (Prediction 1) would be: a substantial proportion of patients invited into DREAM who cannot access imaginal-somatic material despite adherent facilitation, with low completion, low touchstone identification, or facilitators unable to elicit entry even under high-fidelity delivery. Evidence against added benefit (Prediction 2) would be: patients who reliably enter the participatory-imaginal mode but report no perceived benefit, with no corresponding movement on secondary outcome measures or in qualitative interviews; this failure mode would be theoretically informative in its own right, since it would indicate that the phenomenon is real and accessible but that the mechanism linking access to therapeutic benefit is mistaken. Evidence against adequate safety (Prediction 3) would be: adverse events plausibly attributable to the DREAM process itself, such as distress, dissociation, or retraumatization arising specifically during the Amplify or Merge steps at a rate exceeding what other integration methods generally produce; the stopping rules and referral pathways described in Section 5 are, in effect, the operational response to individual instances of this failure mode should it occur. We note that the anecdotes describing spontaneous participatory-imaginal access in the pre-DREAM cohort described in Section 6 do not constitute evidence against these predictions because those patients were never invited into structured re-entry into their psychedelic experience.

If the evidence supports the core predictions, future studies could address more fine-grained hypotheses: that DREAM participants show greater increases in interoceptive awareness than participants receiving standard verbal integration; that the number or quality of touchstones a patient consolidates predicts later integration outcomes; that fidelity to the Amplify and Merge steps specifically correlates with increases in perspectival flexibility; and that patients with higher baseline interoceptive capacity benefit more from the protocol than those with lower baseline capacity. Testing these correlational and moderation hypotheses with any reliability, and testing the comparative prediction at all, requires a sample size and study design beyond what a 10-patient, single-arm feasibility pilot can support. But these predictions do specify the next stage of empirical inquiry.

Which populations are most likely to benefit from DREAM and which psychedelic medications might be best suited for it are important questions for which we do not yet have an empirical answer or clear prediction. Our clinical experience with DREAM as psychedelic integration is in an early phase. After our pilot study and first application to cancer patients we may be able to say more about its suitability to other populations commonly studied in psychedelic-assisted therapy, such as depression, PTSD or substance use disorders. The safety considerations (Section 5) reflect our current judgment about who is at elevated risk, and the broader psychedelic safety literature (62) already underscores the importance of careful participant selection and screening.

## 9 Limitations

Most importantly, while we have specified formal safety procedures, including contraindications, screening, and stopping rules (Section 5), this participatory-imaginal approach is in the early phase of formal evaluation, and there has not yet been an empirical assessment of whether it could be destabilizing for any subgroup of patients. The use of this participatory-imaginal approach in future trials will require facilitator training (63), and whether facilitators can be trained to a manualized version in a timeframe practical for a clinical trial is an open question that the BACK009 pilot is designed to inform. The participants enrolled in our trials to date are self-selected for openness to a group retreat approach, and whether DREAM is workable with patients who do not bring this disposition is unknown. The DREAM protocol likely depends on interoceptive capacity, so a preparation method for training interoception is in preparation (Back, manuscript submitted).

Several additional limitations bear on how the framework and its supporting case material should be interpreted. Robert Bosnak, the originator of Embodied Imagination, is a co-author of this manuscript and supervises facilitator training in our trials, which raises the possibility of allegiance effects in how the method’s promise is characterized and how its fidelity is assessed; a related concern is ordinary facilitator or therapist expectancy effects, since facilitators trained in and committed to this approach could unintentionally shape sessions, or their reports of sessions, toward confirming it. The current pilot studies are not randomized, and because DREAM is delivered within a structured, multi-day group retreat, we cannot yet distinguish effects attributable to DREAM specifically from effects of the retreat or ritual container itself, a question we raise theoretically in Section 6 but have not yet tested empirically. The theoretical vocabulary we draw on (Corbin’s imaginal, Vervaeke’s participatory knowing, Bosnak’s Embodied Imagination) originates in a particular Western philosophical and clinical lineage, and we have not evaluated whether the framework, or the language we use to describe it, generalizes across cultural contexts. We also cannot rule out that some of the vivid imaginal-somatic material patients report reflects suggestibility, or a heightened responsiveness to facilitator expectation or framing, rather than engagement in the process we describe.

Relatedly, participatory-imaginal processing as a construct has not yet undergone formal construct validation: we have not established convergent or discriminant validity against related constructs such as interoceptive awareness, absorption, or dissociation. The falsifiable predictions offered in the Discussion are intended as a first step toward that validation. Also, because DREAM treats imaginal material as psychologically meaningful, there is a risk that vulnerable patients could come to experience such material as literally true as opposed to metaphorically or experientially significant; the safety procedures described in Section 5, including the facilitator’s stance toward imaginal material, are intended to guard against this, but this risk has not been empirically assessed. Finally, our framework and pilot studies are designed for psilocybin. Whether DREAM generalizes to other psychedelic or psychedelic-adjacent medications is unknown, and may depend on each drug’s characteristic phenomenology: agents such as ketamine, whose acute effects can include disembodiment and dissociation rather than intensified interoceptive experience (33), may yield material less suited to somatic re-entry.

## 10 Conclusion

The DREAM protocol is a new method for psychedelic integration designed to engage embodied and imaginal dimensions of psychedelic experiences, and offers a structured framework that can be taught, evaluated for fidelity, and tested empirically. As an alternative to relying on verbal processing, DREAM emphasizes somatic and imaginal engagement as potentially important dimensions. The therapeutic efficacy of the DREAM approach has not been established and is being evaluated. DREAM should therefore be understood not as an established practice for psychedelic integration, but as a structured, clinically plausible, and empirically testable framework for engaging embodied and imaginal dimensions of psychedelic experience.

## Data Availability

The original contributions presented in the study are included in the article/supplementary material. Further inquiries can be directed to the corresponding author.
